# Regulation and Function of Defense-Related Callose Deposition in Plants

**DOI:** 10.3390/ijms22052393

**Published:** 2021-02-27

**Authors:** Ying Wang, Xifeng Li, Baofang Fan, Cheng Zhu, Zhixiang Chen

**Affiliations:** 1 College of Life Sciences, China Jiliang University, 258 Xueyuan Street, Hangzhou 310018, China; wy18865507757@163.com (Y.W.); 19a0902115@cjlu.edu.cn (X.L.); 2 Purdue Center for Plant Biology, Department of Botany and Plant Pathology, Purdue University, 915 W. State Street, West Lafayette, IN 47907-2054, USA; bfan@purdue.edu

**Keywords:** callose, papillae, PMR4, plant cell wall defense, plasmodesmata, plant immunity

## Abstract

Plants are constantly exposed to a wide range of potential pathogens and to protect themselves, have developed a variety of chemical and physical defense mechanisms. Callose is a β-(1,3)-D-glucan that is widely distributed in higher plants. In addition to its role in normal growth and development, callose plays an important role in plant defense. Callose is deposited between the plasma membrane and the cell wall at the site of pathogen attack, at the plasmodesmata, and on other plant tissues to slow pathogen invasion and spread. Since it was first reported more than a century ago, defense-related callose deposition has been extensively studied in a wide-spectrum of plant-pathogen systems. Over the past 20 years or so, a large number of studies have been published that address the dynamic nature of pathogen-induced callose deposition, the complex regulation of synthesis and transport of defense-related callose and associated callose synthases, and its important roles in plant defense responses. In this review, we summarize our current understanding of the regulation and function of defense-related callose deposition in plants and discuss both the progresses and future challenges in addressing this complex defense mechanism as a critical component of a plant immune system.

## 1. Introduction

Callose is a β-(1,3)-D-glucan polysaccharide with some β-1,6-branches that exists in all multicellular green algae and higher plants [1]. In most plants, callose is synthesized by a family of callose synthases and plays important roles in several important biological processes in the plant. During cell division, callose is transiently deposited in the cell plate of the cells undergoing cytokinesis [2,3]. Callose is also a critical component of the transient cell wall surrounding pollen mother cells, the four microspores after meiosis and the pre-cell wall of the growing pollen tube tip [1,4]. Callose is also present in sieve plates, a basic component of the phloem, under normal growing and developmental conditions and can accumulate rapidly and plugs the sieve pores when subjected to stress [5,6]. Similar to this stress response, callose biosynthesis and degradation in the neck region of plasmodesmata help to regulate permeability during abiotic and biotic stresses [7]. In addition, callose is deposited between the plasma membrane and the pre-existing cell wall at sites of pathogen attack [8]. This pathogen-induced callose deposition functions as a chemical and physical defense mechanism for reinforcing plant cell wall and plays an essential role in the defense response to invading pathogens. Over the past 20 years or so, a large number of studies have been published on defense-related callose deposition that address the dynamic nature of pathogen-induced callose deposition, its important roles in plant defense responses, and the complex regulatory mechanisms of defense-related callose deposition. In this review, we summarize these progresses in our understanding of the defense-related callose deposition in plants and discuss both the significance and future challenges of unraveling this complex defense mechanism as a critical component of the plant immune system. 

## 2. Defense-Related Callose Deposition in Plants

Plants are constantly exposed to a large number of microbial pathogens and have evolved complex disease resistance mechanisms that protect plants from pathogens. The first common mechanism of plant disease resistance is through pre-formed structures and compounds such as plant cuticle surfaces, plant cell walls, antimicrobial chemicals and peptides, and other arsenals that inhibit or block pathogen-derived toxins, enzymes, or other activities [9]. The second common disease resistance mechanism is an infection-induced response of the interconnected two-tier immunity system [10]. The first layer is triggered upon recognition of pathogen-associated molecular patterns (PAMPs) by plant plasma membrane-localized pattern recognition receptors (PRRs). Well-characterized PRRs include *Arabidopsis* FLS2 (Flagellin-sensing 2) and EFR (the EF-Tu receptor), which recognize bacterial flagellin and the elongation factor-Tu (EF-Tu), respectively [11]. Adapted pathogens deliver effector proteins to plant cells to suppress PAMP-triggered immunity (PTI) [12]. To counter this, plants have evolved the second layer of the immunity system through recognition of pathogen effectors to activate effector-triggered immunity (ETI). Receptors for ETI are often plant resistance (R) proteins containing nucleotide-binding site (NBS) and leucine-rich repeat (LRR) domains with a Toll-interleukin receptor (TIR) or coiled-coil (CC) N-terminal domain [13]. Both PTI and ETI are associated with diverse signaling processes including dynamic protein interactions and phosphorylation, generation of reactive oxygen species (ROS), Ca^++^ signal spike, and mitogen-activated protein kinase (MAPK) activation [14]. These signaling processes then converge in the nucleus to elicit host transcriptional reprogramming not only for defense but also for balancing defense with growth [15].

In *Arabidopsis*, there are 12 genes encoding GLUCAN SYNTHASE-LIKE (GSL) callose synthases, and pathogen- and PAMP-induced callose deposition is dependent on the GSL5/(POWDERY MILDEW RESISTANT 4 (PMR4) callose synthase [16]. Callose-induced PAMPs include the 22–amino acid sequence of the conserved N-terminal part of flagellin (Flg22) and the 18 amino acid peptide of the bacterial elongation factor EF-Tu (Elf18) [17]. Fungal cell elicitors including chitin, a β-(1,4)-linked polymer of *N*-acetylglucosamine, and chitosan, a randomly distributed β-(1,4)-linked polymer of D-glucosamide and acetylglucosamine, are also potent PAMPs for inducing callose deposition [18]. Besides PAMPs, endogenous damage-associated patterns (DAMP) such as oligogalacturonides from pathogen- or herbivore-damaged plant tissues can function as elicitors to activate callose depositions as well [19]. Pathogen-induced callose can also be primed or enhanced by prior pathogen infection or defense-inducing compounds. For example, induction of systemic acquired resistance (SAR) by local pathogen infection is associated with augmented levels of callose upon secondary pathogen inoculation [20]. Furthermore, resistance-inducing chemicals including salicylic acid (SA), SA analog benzo(1,2,3) thiadiazole-7-carbothioic acid S-methyl ester (BTH), and the nonprotein amino acid BABA can augment depositions of pathogen-inducible callose [21,22]. It has also been recently reported that in tomato and wheat callose priming can be induced by *Rhizophaus irregularis*, a mycorrhizal fungus that can enhance resistance to several pathogens such as the necrotrophic fungal pathogen *Botrytis cinerea* in tomato [23,24]. 

One of the earliest plant defense responses against haustorium-forming pathogens is the deposition of a cell wall- associated apposition called papillae for halting the fungal penetration attempt [8]. If the fungal pathogen is successful in penetration and forms a haustorial feeding structure, the cell wall apposition materials can form collars or neck bands, including partial or even full encasements around the haustorium [25]. Common biochemical constituents of papillae include callose, phenolic, and phenolic conjugates such as phenlic-polyamines, ROS, peroxidases, cell wall structural proteins such as arabinogalactan proteins and hydroxyproline-rich glycoproteins, and cell wall polymers including pectin and xyloglucans [25]. Immuno-fluorescence and immune-gold labeling also found that *Arabidopsis* encasements surrounding the haustoria of the powdery mildew fungal pathogen *Golovinomyces orontii* contain callose, arabinogalactan proteins, rhamnogalacturonanI, a β-linked galactose-containing protein, and xyloglucanin [26], supporting the idea that the papillae and encasements are related and their formation is associated with increased callose deposition.

**Figure 1:**
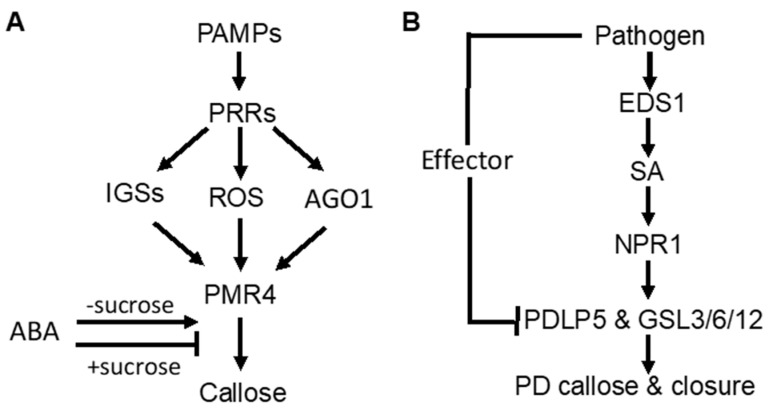
Signaling of pathogen-induced callose deposition in plants. (**A**) pathogen-associated molecular pattern (PAMP)-induced callose deposition is mediated by pattern-recognition receptors (PRRs) and promoted by indole glucosinolates (IGSs), reactive oxygen species (ROS), and RNAi regulatory protein Argonaute1 (AGO1). ABA stimulates but represses PAMP-induced callose deposition in the presence of high sucrose concentrations. (**B**) pathogeninduced callose deposition in plasmodesmata (PD) through the SA-mediated signaling pathway.

Callose deposition is also induced as a defense mechanism in cell wall near the neck zone of plasmodesmata to control their permeability [27]. Plasmodesmata are symplastic junctions between cells that function as important pathways for intercellular communication and molecular exchange, including cell-to-cell spread of viruses [28,29]. The level of callose in the plasmodesmatal neck zone is important for the cell-to-cell movement of cellular molecules and viruses; high levels of callose reduce or even close plasmodesmatal channels, while low levels of callose open them [28,29] (Figure 1). The level of callose in the plasmodesmata is regulated by two groups of enzymes; callose synthases synthesize callose while β-1,3-glucanases degrade it [28,29]. Callose accumulation in the plasmodesmata is induced by many viruses including *Tobacco mosaic virus* (TMV), *Maize dwarf mosaic virus* (MDMV), *Potato virus X* (PVX), and *Tomato bushy stunt virus* (TBSV) [7,28,29,30].

## 3. Regulation of Defense-Related Callose Deposition

### 3.1. Signaling Pathways Controlling Pathogen-Induced Callose Deposition

As part of PTI, PAMP-induced callose deposition is under the control of plant PRRs. Activity of the downstream pathways is marked by common signaling events, such as anion fluxes, protein phosphorylation cascades, accumulation of ROS, and defense gene induction. In *Arabidopsis*, ROS act as positive signals in Flg22-induced callose [31,32] (Figure 1A). The RNAi regulatory protein Argonaute1 also generates various miRNA signals that stimulate or repress Flg22-induced callose [33] (Figure 1A). Flg22-induced callose in *Arabidopsis* also requires intact biosynthesis of 4-methoxylated indole glucosinolates [34] (Figure 1A), suggesting that these secondary metabolites or break-down products play a crucial role in the regulation of callose. Interestingly, Flg22- and chitosan-induced callose differ in the requirement for the NADPH oxidase RBOHD, the glucosinolate regulatory enzymes PEN2, and the callose synthase PMR4 [35]. The *rbohD* mutant accumulated reduced levels of Flg22-induced H_2_O_2_ and failed to deposit enhanced levels of callose upon treatment with Flg22. However, the *rbohD* mutant deposited normal levels of callose in response to chitosan, even though chitosan-induced H_2_O_2_ was reduced. Likewise, the *pen2-2* mutant failed to deposit enhanced callose in response to Flg22, whereas chitosan induced statistically significant enhancements in callose deposition. The *pmr4-1* mutant deposited dramatically reduced levels of basal callose and failed to respond to Flg22. However, chitosan still elicited a residual callose response in *pmr4-1* plants, indicating that one or more other callose synthases than PMR4 are responsible for a portion of chitosan-induced callose. These results demonstrated that PAMP-induced callose is controlled by distinct signaling pathways. 

SA plays an important role in pathogen-induced plasmodesmata closure and associated callose deposition in *Arabidopsis* [36]. Direct exogenous application of SA or bacterial infection induce plasmodesmata closure and callose deposition. SA pathway mutants are impaired in this response. The SA- or pathogen-induced plasmodesmata closure and associated callose deposition requires an ENHANCED DESEASE RESISTANCE1(EDS1)– and NONEXPRESSOR OF PATHOGENESIS-RELATED GENES1 (NPR1)–dependent SA pathway and is dependent on the regulator of plasmodesmal gating PLASMODESMATA-LOCATED PROTEIN5(PDL5) [36](Figure 1B). These results indicate that SA is a crucial signal in the regulation of cell-to-cell communication and trafficking via plasmodesmata in plant cells during innate immune responses. 

Abscisic acid (ABA) signaling has an important effect on induced or primed callose deposition. Callose priming induced by the nonprotein amino acid β-amino butyric acid (BABA) requires an intact ABA-dependent pathway in *Arabidopsis*. BABA-induced resistance in *Arabidopsis* against *Plectosphaerella cucumerina* is known to be mediated by callose priming. Indole-3-carboxylic acid (ICOOH, also known as I3CA) mediates BABA-induced resistance in *Arabidopsis* against *P. cucumerina*. I3CA treatment increased ABA levels, which activates a starch amylase (BAM1) to trigger augmented callose deposition against *P. cucumerina* [37]. A similar role of ABA and starch metabolism has also been reported in mycorrhiza-induced callose priming in tomato plants upon infection by necrotrophic fungal pathogen *B. cinerea* [24]. Intriguingly, ABA can enhance or repress Flg22-induced callose deposition in *Arabidopsis* depending on growth conditions [35] (Figure 1A). Under most growth conditions, ABA has a stimulatory effect on Flg22-induced callose deposition. However, ABA reduced basal and PAMP-induced callose at low light intensity, high sucrose concentration and vitamins (Figure 1A). ABA also represses Flg22-induced callose in hydroponic *Arabidopsis* grown with the a relatively high concentration of sucrose in the growth medium (0.5%) [34,35] (Figure 1A). These results suggest that ABA’s role in defense-related callose deposition and priming is influenced by carbohydrate metabolism. 

### 3.2. Biogenesis and Activation of Pathogen-Responsive Callose Synthases

**Figure 2:**
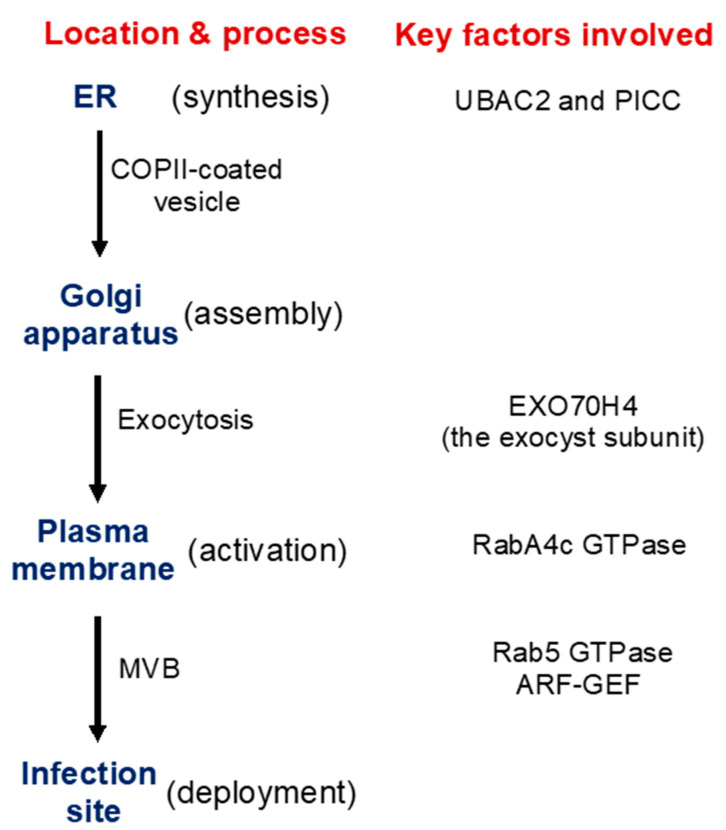
Synthesis, transport, activation, and recruitment of *Arabidopsis* PMR4 callose synthase during plant defense responses. Some of the important factors identified to be involved in these processes are indicated.

As a large transmembrane protein, GSL5/PMR4 belongs to the family of glucan synthases that include plant cellulose synthases. Plant cellulose synthases are synthesized on the endoplasmic reticulum (ER) and go through the ER quality control system for proper folding and modification before being assembled into complexes in the Golgi apparatus and transported to the plasma membrane through the Golgi- and post-Golgi trafficking system [38]. Many proteins involved in plant immune responses, including pattern-recognition receptors and extracellular defense-related proteins, are also synthesized on the ER, go through the ER quality control, and ultimately are transported to their destinations through the secretory pathway. We have recently shown that two *Arabidopsis* homologs of UBAC2, a conserved ER protein implicated in ER quality control and ER-associated degradation (ERAD), play a critical role in pathogen- and PAMP-induced callose deposition [39]. The UBAC2 proteins interact with the plant-specific PAMP-INDUCED COILED COIL (PICC) protein, which is also localized in the ER and is important for pathogen- and PAMP-induced callose deposition (Figure 2). Therefore, the evolutionarily conserved UBAC2 and plant-specific PICC proteins are critical components of an ER pathway with an important role in plant immunity by regulating pathogen-induced callose deposition. The compromised phenotypes of the *ubac2* and *picc* mutants in PTI and pathogen/PAMP-induced callose deposition could be rescued by overexpression of *PMR4*, suggesting that disruption of *UBAC2* and *PICC* genes may reduce the accumulation of PMR4 callose synthase in the *ubac2* and *picc* mutants, leading to defects in pathogen/PAMP-induced callose deposition [39]. Further analysis using both GFP- and myc-labeled PMR4 indeed discovered that disruption of *UBAC2* or *PICC* reduced the levels of plasma-membrane-localized PMR4 [39]. These results indicate that the evolutionarily conserved UBAC2 proteins function in coordination with a plant-specific protein PICC in the positive regulation of the biogenesis of PMR4, either by stabilizing the callose synthase or degrading a negative regulator of PMR4 stability (Figure 2). 

In addition, there is evidence for PMR4 activation in response to pathogen infection or other stress signals. In uninfected *PMR4*-overexpression lines, no significant increase in the callose synthase activity or callose deposition was observed [40]. In yeast, activation and translocation of callose synthases involve GTPases [41,42]. In *Arabidopsis*, PMR4 interacts with GTPase RabA4c [43] (Figure 2). Similar to PMR4 overexpression, RabA4c overexpression leads to complete penetration resistance to the virulent powdery mildew fungal pathogen in a PMR4-dependent manner [43]. By contrast, overexpression of a dominant negative form of RabA4c fails to increase callose deposition or penetration resistance [43]. These results indicate that the RabA4c GTPase plays a critical role in the activation and translocation of PMR4 during pathogen-induced callose deposition. As a transmembrane protein synthesized on the ER, PMR4 is likely subjected to additional regulatory mechanisms for its biogenesis, trafficking, and activation to ensure biosynthesis of callose in a timely manner. 

### 3.3. Transport Processes in Pathogen-Induced Callose Deposition

Exocytosis is a process by which cells are able to not only move materials from within a cell to the exterior of the cell, but also insert membrane proteins (such as ion channels and cell surface receptors), lipids, and other components into the cell membrane [44]. Vesicles containing these membrane components fully fuse with and become part of the plasma membrane. The exocyst, an octameric protein complex involved in exocytosis, tethers and spatially target post-Golgi vesicles to the plasma membrane prior to vesicle fusion [44]. It has been demonstrated that the exocyst subunit EXO70H4 is important in the deposition of the callose-rich cell wall layer for biogenesis of the trichome secondary cell wall [45,46]. PMR4 is a callose synthase responsible for the synthesis of callose in the trichome and PMR4 colocalizes with EXO70H4 on plasma membrane microdomains that do not develop in the *exo70h4-1* mutant. Significantly, *EXO70H4* expression is induced by pathogen elicitor Flg22, which induces callose deposition, in epidermal pavement cells [46]. These results raise a strong possibility that EXO70H4-dependent exocytosis is also involved in the trafficking of newly synthesized PMR4 callose synthase proteins from the Golgi apparatus to the plasma membrane (Figure 2).

Another transport process important for defense-related callose deposition is the recruitment of callose synthases protein such as PMR4 in *Arabidopsis* from the plasma membrane and transport in vesicle-like bodies to the site of attempted penetration after fungal infection (Figure 1). There is strong evidence that multivesicular bodies (MVBs) are involved in the transportation and delivery of defense components to the forming papilla. Studies have showed accumulation of MVBs and cell wall-associated paramural bodies (PMBs) in the vicinity of pathogen-induced papillae [47,48,49]. PMBs, which are situated between the cell wall and the plasma membrane, are likely to have resulted from the fusion of MVBs with the plasma membrane [50]. Plant MVBs and PMBs have been observed near papillae in plant cells infected by pathogenic fungal, bacteria and nematodes for delivery of defense-related molecules including phytoalexins, callose and ROS to papillae [47,48,51,52,53]. In addition, in plant interaction with filamentous pathogens, each haustorium formed upon successful penetration is surrounded by the plasma membrane of the plant cell termed extrahaustorial membrane (EHM), which is likely synthesized de novo [54]. In tobacco (*Nicotiana benthamiana*) cells invaded by oomycete pathogen *P. infestans*, the Rab7 GTPase RabG3c MVB marker protein, but not a tonoplast-localized sucrose transporter, is recruited to the EHM [55]. In *Arabidopsis*, the Rab5 GTPase, also an MVB marker, accumulates in the EHM after infection with a powdery mildew fungus [56] (Figure 2). Thus, specific rerouting of MVBs from the vacuole to the host-pathogen interface may participate in the formation or modulation of the EHM. In barley, MVBs contained the ADP-ribosylation factor (ARF) GTPase ARFA1b/1c that was important for callose deposition at powdery mildew penetration sites [51]. RNAi knockdown or expression of a dominant negative ARFA1b/1c variant abolished callose accumulation at penetration sites and resulted in increased fungal penetration success. ARFA1b/1c localized to an endosomal MVB compartment that accumulated at fungal penetration sites prior to the accumulation of callose. Interestingly, the *Arabidopsis* ARF-GEF (guanine nucleotide exchange factor) MIN7 is required for normal levels of callose deposition in response to the *Pseudomonas syringae* pv. *tomato* ΔCEL mutant, suggesting that an ARF-dependent vesicle trafficking process may also play a role in callose deposition at sites of pathogen detection in *Arabidopsis* [57] (Figure 2).

## 4. Function of Callose Deposition in Plant Defense

### 4.1. Effects of Callose Deposition on Plant Cell Wall

It has been proposed that callose polymers can reinforce the cell wall structure by increasing its stiffness at the site of infection to restrict the ingression of pathogen-secreted cell wall-degrading enzymes [1]. In a recent study, the molecular interactions and physico-mechanical properties of the mixtures of callose and cellulose were analyzed in ionic liquid solution and hydrogels using a combination of atomic force and scanning electron microscopy, nuclear magnetic resonance and Fourier Transform InfraRed (FTIR) spectroscopy [58]. Intriguingly, the study discovered that addition of callose reduced the stiffness of cellulose hydrogels, suggesting that callose has the capacity to increase the resilience of cellulosic materials to large deformations [58]. Therefore, the callose properties and its potential role as cellulose modifier in plant cell wall are still not fully understood.

Comparative analysis using localization microscopy of *Arabidopsis* wild-type and transgenic plants overexpressing the pathogen-induced callose synthase *PMR4* has provided additional information about the distribution and architectures of callose at the sites of papillae formation and helped visualize the three-dimensional structure of callose deposition at sites of attempted fungal penetration [59]. Using aniline blue for staining callose and pontamine fast scarlet 4B for the overlying cellulosic cell wall, these authors observed a migration of callose fibrils into the cell wall. In wild-type *Arabidopsis* leaves, only single callose fibrils migrated from the dense callosic core of the papilla into and penetrated through the cellulosic cell wall. On the other hand, a dense collection of callose/cellulose fibrils was observed along the papilla core region and the lateral field of callose in epidermal leaf cells of *PMR4*-overexpressing plants at the sites of attempted penetration by the powdery mildew fungus. In addition, there is a callose layer on the surface of the pre-existing cellulosic cell wall facing the invading pathogen. This additional callose layer provided enhanced resistance to cell wall degrading enzymes [59]. Therefore, callose deposition may not only strengthens the cell wall but also functions as a sealant, a load-bearing structure, or a matrix for the deposition of other cell wall components. 

### 4.2. Association of Increased Callose Deposition with Plant Disease Resistance

**Figure 3:**
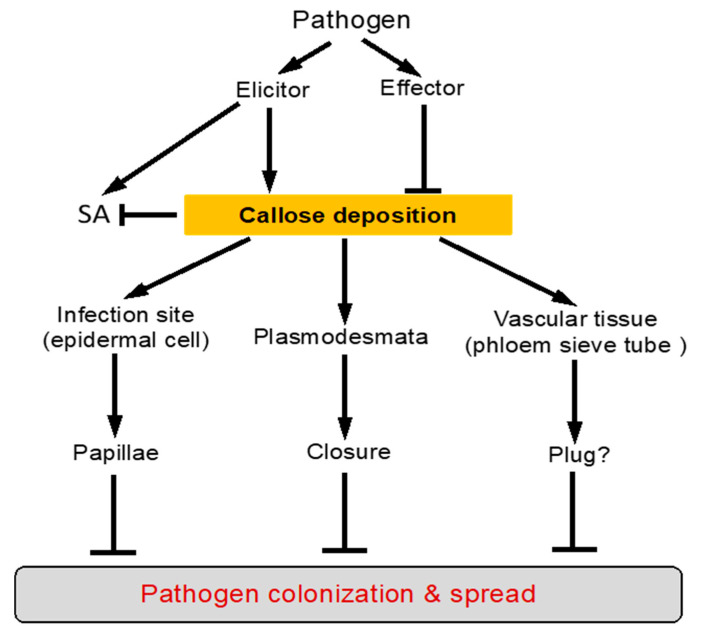
Induction and roles of defense-related callose deposition in plants. Pathogen elicitor-activated signaling of plant innate immune responses leads to increased callose deposition at the sites of pathogen attack, at plasmodesmata and in the vascular tissues. Formation of callose-rich papilla at the infection sites helps restrict penetration and colonization by invading pathogens. Increased callose deposition at plasmodesmata leads to plasmodesmata closure, which helps restrict pathogen spread. Increased callose deposition in the vascular tissues such as phloem sieve tubes could also functions as a defense mechanism for reducing the colonization and transport of vascular pathogens. In *Arabidopsis*, pathogen-induced SA signaling is negative regulated by PMR4-dependent callose deposition. Pathogens contain effector proteins that inhibit or block defense-related callose deposition as counter-defense mechanisms.

Even though the exact effects of callose on physico-mechanical properties of plant cell wall are not fully clear, the biological evidence, both direct and indirect, for a critical role of callose in plant defense is extensive and compelling. Apart from the inducible nature of callose deposition by pathogens as discussed earlier, there are a large number of reported studies showing associations between callose deposition and increased resistance to invading pathogens (Figure 3). In the well-studied interaction between barley and the powdery mildew fungus *Blumeria graminis* f. sp *hordei*, barley papillae from incompatible interactions have significantly higher accumulation of callose, as well as thionin proteins, the phenolic conjugate p-coumaroyl-hydroxyagmatine, and ROS than barley papillae from compatible interactions [60]. The overall susceptibility and resistance of a barley genotype to the powdery mildew fungal pathogen is determined by the percentage of papillae that stop penetration effectively (nonpenetrated or effective papillae) to the papillae that are ultimately penetrated by the fungus (penetrated or ineffective papillae). To determine which components are responsible for making papillae an effective barrier to fungal penetration, Chowdhury et al. have further compared the composition of papillae in susceptible cells with that of papillae in resistant cells, within the same barley line [60]. The study revealed higher concentrations of callose, arabinoxylan and cellulose in effective papillae, than in ineffective papillae. The papillae have a layered structure, with callose and arabinoxylan primarily in the inner core and arabinoxylan and cellulose in the outer layer [60]. Callose deposition at the plasmodesmata is also associated with the defense responses of host plants against viral infection. In the incompatible interaction between soybean and *Soybean mosaic virus* (SMV), callose deposition at the plasmodesmata was readily detected at the sites of inoculation but viral RNA of coat protein (CP-RNA) was undetectable by RT-PCR in the leaf above the inoculated one (the upper leaf) [61]. In the compatible interaction, however, callose deposition at the plasmodesmata was not detected at the site of infection but the viral CP-RNA was detected by RT-PCR in the upper leaf [61].

Plant defense can be primed chemically or biologically for faster and/or stronger activation upon subsequent challenge by a microbial pathogen. Studies have shown that callose deposition is also associated with plant defense priming. In *Arabidopsis*, BTH is a synthetic inducer of SAR, which is also associated with defense priming [21]. BTH itself was essentially inactive at the immediate induction of *PHENYLALANINE AMMONIA-LYASE* (*PAL*) gene activation and callose deposition. However, pretreatment with BTH greatly augmented the subsequent *PAL* gene expression induced by *P. syringae* pv. *tomato* infection, wounding, or water infiltration. BTH pretreatment enhanced the callose deposition induced by wounding or water infiltrating [21]. Enhanced *PAL* gene activation and callose biosynthesis were found in unpretreated *Arabidopsis constitutive expresser of pathogenesis-related* genes *1* and *5* (*cpr1* and *5*) mutants, which display constitutive SAR. Increased callose deposition is also associated with mycorrhiza-induced resistance [21]. In tomato plants, for example, mycorrhizal tomato plants inoculated with *R. irregularis* displayed callose priming upon *B. cinerea* infection in correlation with increased resistance to the necrotrophic fungal pathogen [24]. 

### 4.3. Chemical Inhibition of Callose Synthesis Compromises Plant Disease Resistance

The most direct evidence for a critical role of callose deposition in plant defense comes from the studies through chemical, genetic or molecular manipulation of callose synthesis or degradation in plant cells and analysis of the impacts on plant disease resistance. Chemical manipulation of callose deposition is often achieved through chemical inhibition of callose synthesis by chemical inhibitors such as 2-deoxy-D-glucose. This approach has been used in the early analysis of the role of callose in the resistance to powdery mildew in the *mlo* mutant barley lines, which is characterized by a high frequency of early formed, callose-containing papillae [62]. Treatment of *ml*-*o* resistant barley coleoptiles with 10^−5^ m 2-deoxy-D-glucose decreased papilla frequency from 92% to 41% and increased penetration efficiency from 15% to 78%. Inhibition of callose synthesis by 2-deoxy-D-glucose delayed papilla formation and sites with late-forming papillae were penetrated by the fungus, whereas sites with early-forming papillae were not [62]. These results indicated that early papilla formation is the mechanism of *mlo* resistance and callose formation facilitates papilla deposition in this *mlo* barley mutant (Figure 3).

A similar approach has been used to test the role of callose deposition in interaction between soybean and SMV. As discussed earlier, in the incompatible interaction between soybean and SMV, soybean resistance to the virus is associated with callose deposition at the plasmodesmata. Injection of 2-deoxy-D-glucose inhibited the fluorescence due to callose formation at the inoculation point in the incompatible combination [61]. At the same time, necrosis was developed and the viral CP-RNA was detected in the upper leaf and characteristics of hypersensitive response (HR) were observed at the inoculation sites in the incompatible combination [61]. Thus, during the incompatible interaction of soybean to viral infection, callose deposition at plasmodesmata plays a critical role in restricting the movement of the virus between cells and this callose-mediated action occurs prior to the HR response. Likewise, in tomato plants, inoculation with *R. irregularis* promoted resistance to the necrotrophic fungal pathogen *B. cinerea* and these mycorrhizal tomato plants displayed callose priming upon *B. cinerea* infection. The callose inhibitor 2-deoxy-D-glucose inhibited callose priming and abolished mycorrhiza-induced resistance, confirming the relevance of callose in the bioprotection [24]. While the chemical inhibition of callose synthesis provides a simple approach to assess the role callose in plant defense, it has the problem of potential nonspecific inhibition of other enzymes caused by the inhibitors. In addition, callose synthases are encoded by a multi-gene family and this approach cannot assign the critical role of callose synthesis to specific callose synthases.

### 4.4. Genetic and Molecular Evidence for the Role of Callose Synthesis in Plant Disease Resistance

Although callose deposition in papillae at sites of fungal penetration has been widely recognized as early response of host plants to microbial attack that impedes entry of the fungus, knockout of *Arabidopsis GSL5* depleted callose from papillae but only marginally enhanced the penetration of the grass powdery mildew fungus *B. graminis* on the nonhost *Arabidopsis* [16]. The *Arabidopsis gsl5* mutant plants also respond normally to the virulent bacterial pathogen *P. syringae*. Even more surprisingly, *Arabidopsis powdery mildew resistant 4* (*pmr4*) mutants, which resulted from a mutation in the *GSL5* gene, lack pathogen-induced callose deposition but become resistant to several normally virulent powdery mildew species and *Peronospora parasitica* [16,63]. Likewise, RNA silencing or mutations of tomato *PMR4* gene also enhanced resistance against the powdery mildew pathogen *Oidium neolycopersici* [64,65]. These results are unexpected, given the large body of evidence that supports a critical role of callose in plant defense.

Further analysis has revealed two major processes that complicate the evaluation of the role of callose deposition in plant disease resistance. First, as will be detailed in the following section, pathogen-induced callose deposition is targeted and inhibited by pathogen effectors. For example, a number of effectors from *P. syringae* are suppressors of pathogen-induced callose deposition and as a result, while the *Arabidopsis pmr4* mutant is normal in response to virulent *P. syringae*, it supports 20-fold more growth than wild-type (WT) plants of a strain of *P. syringae* that is deficient in the secretion system for delivery of the effector proteins and, therefore, is unable to suppress pathogen-induced callose deposition [66] (Figure 3). Overexpression of *PMR4* in transgenic *Arabidopsis* plants elevates early callose deposition and confers complete restriction to penetration by powdery mildew fungal pathogen [40], further supporting the critical role of increased callose deposition in plant disease resistance. The second complication for evaluating the role of PMR4-dependent callose is the finding that mutation of *PMR4* is associated with hyper-activation of SA signaling, leading to increased resistance to powdery mildew fungal pathogens [63] (Figure 3). This finding indicates an unexpected role of PMR4-dependent callose deposition in the suppression of SA-mediated defense responses. Therefore, PMR4-dependent callose deposition has complex roles in plant defense against pathogen invasion.

Even though the polysaccharide composition of papillae deposited at the barley cell wall during infection by the powdery mildew pathogen, *B. graminis* f. sp. *hordei* was well characterized, the role of callose in papilla-based penetration resistance in the barley-powdery mildew fungus was unclear because the genes involved in the observed callose accumulation were not identified. Through both comparative and functional genomics approaches, Chowdhury and colleagues identified the barley glucan synthase-like 6 (HvGSL6) to be the functional orthologue of *Arabidopsis* GSL5 in the barley genome [67]. HvGSL6 is the only pathogen-induced gene among the HvGSLs examined and encodes a protein with the highest sequence identity to *Arabidopsis* GSL5. Most importantly, down-regulation of HvGSL6 through double-stranded RNA interference (dsRNAi)-mediated silencing was associated with reduced accumulation of papillary and wound callose and increased susceptibility to penetration of the papillae by the powdery mildew fungal pathogen [67]. The results indicate that callose deposition plays a critical role in the barley fungal penetration resistance mechanism (Figure 3).

There is also strong molecular and genetic evidence for a critical role of plasmodesmatal callose in restricting cell-to-cell movement of viral pathogens (Figure 3). In tobacco, antisense silencing of a class I β-1,3-glucanase (GLU I), which degrades callose, led to reduced susceptibility to virus infection [30]. These antisense transgenic plants exhibited delayed intercellular trafficking via plasmodesmata of *Tobacco mosaic virus* (TMV)), *Potato virus X* (PVX), and the movement protein of *Cucumber mosaic virus* (CMV). Based on the cell-to-cell movement of dextrans and peptides determined by a novel biolistic method, the plasmodesmatal size exclusion limit (SEL) of the antisense plants was reduced from 1.0 to 0.85 nm [30]. Therefore, downregulation of GLU I expression had a broad effect on plasmodesmatal movement that affect multiple types of viruses. These GLU I-silenced plants also has increased deposition of callose in response to 32 °C treatment, treatment with the fungal elicitor xylanase, and wounding [30]. These results strongly suggest that GLUI has an important role in callose turnover, which, in turn, regulates plasmodesmatal SEL and movement of viral pathogens. In *Arabidopsis*, the β-1,3-glucanase AtBG_pap is associated with plasmodesmata [68]. In *atbg_pap* mutants, callose at the plasmodesmata was increased and virus spread was reduced [68]. These results further support the idea that the plasmodesmata-associated β-1,3-glucanase is directly involved in regulation of callose at plasmodesmata and cell-to-cell spread of viruses in *Arabidopsis*.

### 4.5. Undermining Callose-Mediated Defense by Pathogen Effectors

The proposed role of callose deposition in plant immune responses is supported by its inhibition by many defense-suppressing virulence effectors from different types of microbial pathogens (Figure 3). In gram-negative bacterial pathogens, bacterial effector proteins secreted through the type III secretion system (TTSS) play a crucial role in causing plant and human diseases by suppressing host immune responses [69]. It was demonstrated several decades ago that TTSS-deficient mutants of *Xanthomonas campestris* pv. *vesicatoria* and *P. syringae* pv. *phaseolicola*, as well as a saprophytic bacterium, cause the plant cell wall to thicken, forming a papilla [70,71,72]. By contrast, the TTSS-competent wild-type *X. campestris* pv. *vesicatoria* does not induce papillae formation. In *Arabidopsis*, the TTSS-deficient *hrcC* strain of *P. syringae* pv *tomato* DC3000 induced abundant callose deposition while the virulent wild-type strain of the same bacterial pathogen did not. Through a combination of large-scale host gene expression profiling, transgenic expression of a DC3000 effector, and cytological examination, Hauck and colleagues have identified AvrPto as a suppressor of the papillae-associated cell wall defense including callose deposition [73]. Transgenic expression of AvrPto in *Arabidopsis* plants repressed a similar set of host genes, compromised defense-related callose deposition in the host cell wall, and permitted substantial multiplication of an TTSS-deficient strain of *P. syringae*. These results indicate that AvrPto is one of the bacterial effectors that suppresses cell wall-based defense [73]. Similar suppressors of plant cell wall defense are also present in fungal pathogens. In plant-fungal interaction, haustorial encasements appear to be defensive structures rather than structures that accommodate haustorium formation because they were not observed around haustoria incompatible interactions, suggesting that adapted fungi have the ability to suppress formation of encasements. Encasements were observed around ~20% of *G. orontii* haustoria in *Arabidopsis*, but were not observed around *G. cichoracearum* haustoria, suggesting that *G. cichoracearum* can suppress this defense response more effectively than *G. orontii* [52,74]. Understanding how haustorium-forming pathogens suppress the formation of encasements and defeat papilla-associated cell wall defenses will be a significant challenge for future research.

Microbial pathogens also target callose deposition at the plasmodesmata to promote their cell-to-cell trafficking. Plant genes encoding β-1,3-glucanase are known to be induced in response to viral infection, which would promote callose degradation at the plasmodesmata and facilitate movement protein-driven virus spready. Specific pathogen effectors have also been identified that undermine plasmodesmatal callose deposition. *Potato virus* Y (PVY) is a major pathogen for potato production due to the increasing popularity of strain-specific resistant cultivars. In many potato cultivars, such as cultivar Premier Russet (PR), local hypersensitive response (HR) at the site of infection protects against the most common PVY strain PVY^O^, but often fails to restrain systemic spread of necrotic PYV^N^ strains [75]. Callose accumulation plays a role in the strain-specific resistance responses to PVY infection. All PVY strains through the viral-encoded helper component proteinase (HCPro) proteins are naturally capable of suppressing pathogenesis-related callose formation in a susceptible host, which can be dissociated from viral replication [75]. However, unlike the necrotic strain, the common PVY strain PVY^O^ and its corresponding HCPro^O^ are unable to block callose accumulation in resistant potato hosts, in which there is an abundant callose deposition and the inability of the virus to spread. The substitution of eight amino acid residues within the HCPro C-terminal region that differ between PVY^O^ and PVY^N^ strains and were responsible for eliciting the HR response, are sufficient to restore the ability of HCPro^O^ to suppress callose accumulation even in resistant hosts [75].

The RxLR3 effector of the plant pathogen *Phytophthora brassicae* is also inhibitory to plant callose deposition [76]. Co-immunoprecipitation followed by liquid chromatography-tandem mass spectrometry identified three closely related members of the *Arabidopsis* callose synthase family, GSL3, GSL6, and GSL12 as targets of RxLR3. RxLR3 co-localized with the plasmodesmatal marker protein PDLP5 (PLASMODESMATA-LOCALISED PROTEIN 5) and with plasmodesmata-associated callose deposits. RxLR3 reduced callose depositions and promoted cell-to-cell trafficking, supporting the effectors as an inhibitor of plasmodesmatal callose synthases [76]. These results support a virulence function of the RxLR3 effector *P. brassicae* as a positive regulator of plasmodesmata transport for competition with *Arabidopsis* hos for control of cell-to-cell trafficking.

## 5. Conclusions and Prospects

Callose as the most prominent cell wall component of papillae was first reported more than 120 years ago and has received extensive attention in the research on the physical and chemical basis of plant disease resistance. Over time, a large body of evidence has established the formation of callose-rich papillae as one of the earliest plant defense responses at the cellular level in all plants following infection by different types of pathogens. Over the past 20 years or so, molecular and genetic approaches have established the important and complex roles of defense-related callose deposition in plant immune responses against microbial pathogens (Figure 3). Molecular mechanisms that control the synthesis, activation, and transport of defense-responsive callose synthases and callose polymers are also starting to emerge (Figure 2). Despite this progress, there are still many important questions remained to be addressed in order to fully understand the roles of defense-related callose deposition in plant immune responses. First, even though it has been long thought that callose can reinforce plant cell wall by increasing its stiffness at the site of infection to restrict the ingression of pathogens, physicochemical evidence for the nature of callose effects on plant cell wall is still lacking. In particular, how callose interacts with other components of plant cell wall such as cellulose to alter or even generate new functional properties of plant cell wall would be highly valuable for understanding the biological roles of callose deposition in plants. Second, a vast majority of studies on defense-related callose deposition have been focused on callose associated with pathogen-induced papillae normally in the pavement cells of plant leaves and at the plasmodesmata, both of which play important roles in restricting pathogen ingression and spread. However, there are other plant tissues such as vascular tissues which many plant pathogens are associated with or even confined to, but little information is available on whether callose deposition plays a similarly important role in plant defense against attempted pathogen invasion in these special tissues. A recent study has reported that in citrus, infection by the phloem-limited fastidious protepbacterium *Canditatus Liberibacter asiaticus*, which is transmitted by the Asian citrus psyllid (*Diaphorina citri*) was associated with up-regulated expression of specific citrus callose synthase genes, increased deposition of callose in the phloem sieve tubes, and accumulation of starch in the leaves [77]. It will be of high interest to determine whether induced callose deposition in the phloem functions as a defense mechanism for reducing the bacterial colonization via phloem and causing starch accumulation in the leaves due to inhibited phloem transport (Figure 3). Third, one of the most important discoveries in the functional analysis of defense-related callose deposition is the hyperactivation of SA signaling in the *pmr4* mutant in *Arabidopsis*. This unexpected role of the callose synthase in plant defense signaling has also been found in tomato but apparently not in barley. Further research will be necessary to confirm whether this is indeed plant species-specific and, if so, what is the molecular basis for the crosstalk between callose- and SA-mediated defense pathways. Fourth, in contrast to the very early recognition of the dynamic, rapid, and focal nature of defense-related callose deposition, our understanding of the regulatory mechanisms for the synthesis and transport of pathogen-induced callose is very limited. A better knowledge about the function and regulation of defense-related callose deposition in plants will provide important new insights into the molecular basis of plant immunity and help develop new strategies for generating disease-resistant crop plants.
